# Supplementary material for: Cationic Pillar[6]arene Induces Cell Apoptosis by Inhibiting Protein Tyrosine Phosphorylation Via Host–Guest Recognition
Source: Int J Mol Sci. 2020 Jul 15;21(14):4979. doi: 10.3390/ijms21144979 (PMC7404071; doi:10.3390/ijms21144979)
Supplement: Supplementary file 1 [file ijms-21-04979-s001.pdf]

International Journal of *Molecular Sciences*

MDPI

Supplementary Materials

Cationic Pillar[6]arene Induces Cell Apoptosis by Inhibiting Protein Tyrosine Phosphorylation via Host-Guest Recognition

Can-Peng Li [1,†], Yu-Xun Lu [2,†], Cheng-Ting Zi [3,†], Yu-Ting Zhao [1], Hui Zhao [2,*] and Ya-Ping Zhang [2,4,*]

1 Key Laboratory of Medicinal Chemistry for Natural Resource, Ministry of Education, School of Chemical Science and Technology, Yunnan University, Kunming 650091, China; lcppp1974@sina.com (C.-P.L.) ; zyt94817@163.com (Y.-T.Z.)

2 State Key Laboratory for Conservation and Utilization of Bio-resource, School of Life Science, Yunnan University, Kunming 650091, China; l273581722@163.com (Y.-X.L.)

3 State Key Laboratory of Pu-er Tea Science, Ministry of Education in Yunnan, College of *State* Science, Yunnan Agricultural University, Kunming, 650201, China; chengtingzi@163.com (C.-T.Z.)

4 Key Laboratory of Genetic Resources and Evolution, Kunming Institute of Zoology, Chinese Academy of Sciences, Kunming 650223, PR China

† These authors contributed equally to this work

* Correspondence: zhaohui@ynu.edu.cn (H.Z.); zhangyp@mail.kiz.ac.cn (Y.-P.Z.)

1. Synthesis and characterization of pillararenes

1.1. Synthesis and characterization of cPA6

The cationic pillar[6]arene (cPA6) and cationic pillar[5]arene (cPA5) were synthesized according to published procedures[1-2] and their synthetic route is shown in Scheme S1.

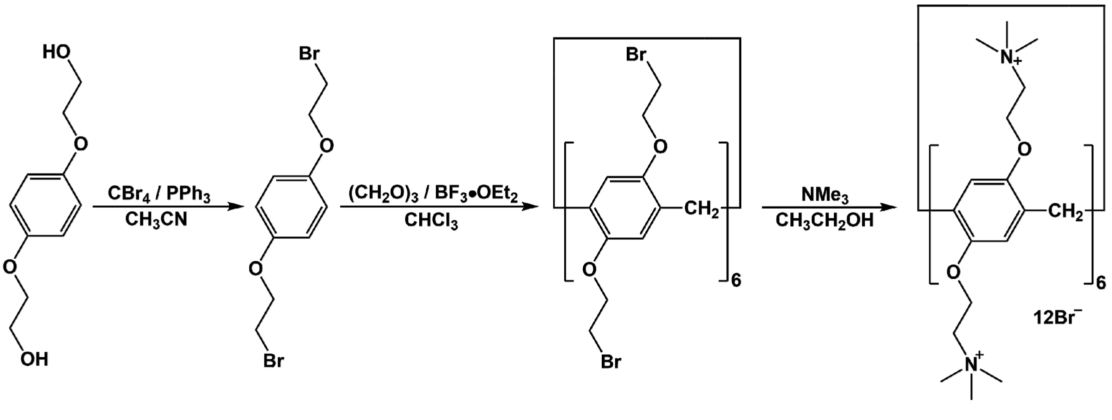

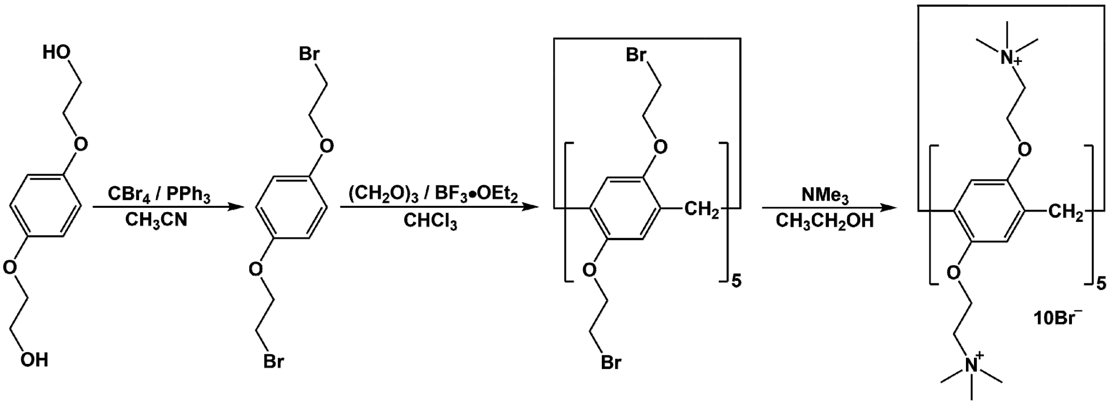

Scheme S1. Synthetic route to cPA6 and cPA5.

1.1.1. Synthesis of compound 1

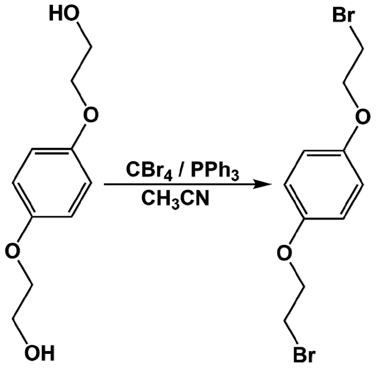

Scheme S2. Synthetic route to 1.

Carbon tetrabromide (39.8 g, 120 mmol) was slowly added to a solution of 1,4-bis(2-hydroxyethoxy)benzene (10.0 g, 50.4 mmol) and triphenylphosphine (31.5 g, 120 mmol) in 250 mL of dry acetonitrile with violent stirring. The reaction mixture was stirred for 4 h under N_2 at room temperature. Then, 200 mL of cold water was added to the reaction mixture, where product **1** precipitated as a white solid. The product was collected by vacuum filtration, thoroughly washed with methanol/water (3:2), and then recrystalized from methanol. The white flake-like crystals were dried under high vacuum (15.2 g, 93 %). The ^{1}H NMR and ^{13}C NMR spectra of **1** are shown in **Figure S1**. ^{1}H NMR (400 MHz, chloroform-*d*, room temperature) δ (ppm): 6.864 (s, 4H), 4.247 (t, J = 6.4 Hz, 4H), and 3.618 (t, J = 6.4 Hz, 4H). ^{13}C NMR (100 MHz, chloroform-*d*, room temperature) δ (ppm): 152.79, 116.06, 68.68, and 29.28.

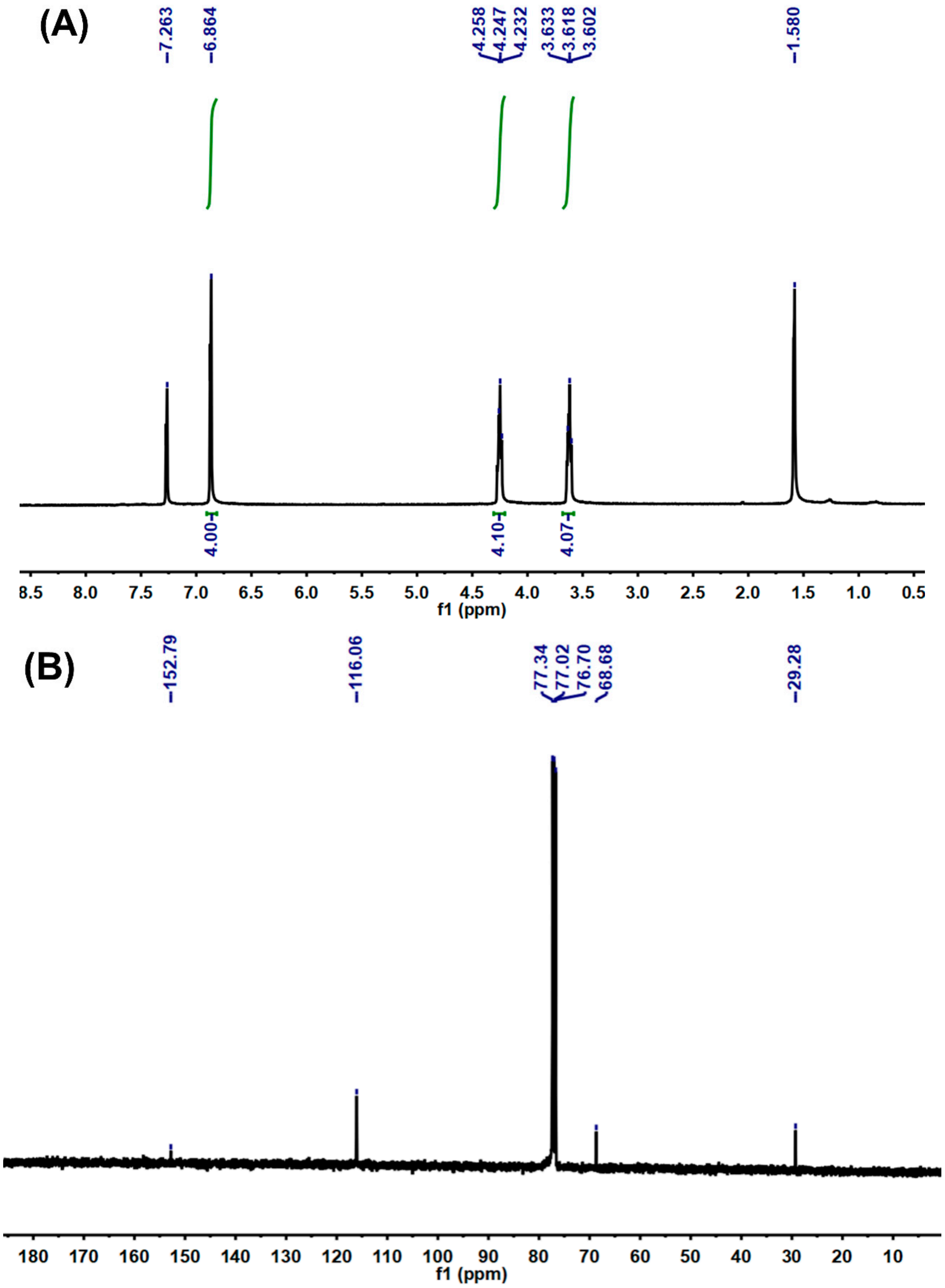

Figure S1. (**A**) ^{1}H NMR (400 MHz, chloroform-*d*, room temperature) and (**B**) ^{13}C NMR spectra (100 MHz, chloroform-*d*, room temperature) of 1.

1.1.2. Synthesis of Br6

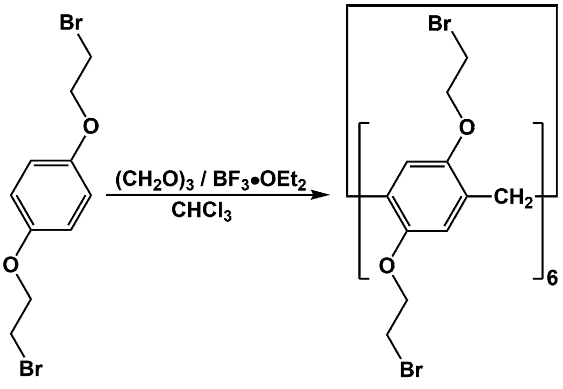

Scheme S3. Synthetic route to Br6.

To a solution of 1 (5.0 g, 15.4 mmol) in chloroform (300 mL), 1,3,5-trioxacyclohexane (0.92 g, 30.8 mmol) was added under nitrogen atmosphere. Then, boron trifluoride diethyl etherate (BF3·OEt2, 4.38 g, 30.8 mmol) was added to the solution and the mixture was stirred at room temperature for 3 h. After the reaction was stopped by water and the solvent was removed. Then the obtained solid was purified by column chromatography on silica gel with petroleum ether/dichloromethane (1:1 v/v) as the eluent to obtain a white powder (2.1 g, 43 %). The 1H NMR and 13C NMR spectra of Br6 are shown in Figure S2. ^{1}H NMR (600 MHz, chloroform-d, room temperature) δ (ppm): 6.781 (s, 12H), 4.163 (t, J = 5.6 Hz, 24H), 3.869 (s, 12H), and 3.553 (t, J = 5.6 Hz, 24H). ^{13}C NMR (150 MHz, chloroform-d, room temperature) δ (ppm): 150.18, 128.52, 115.84, 68.97, 30.65, and 30.31.

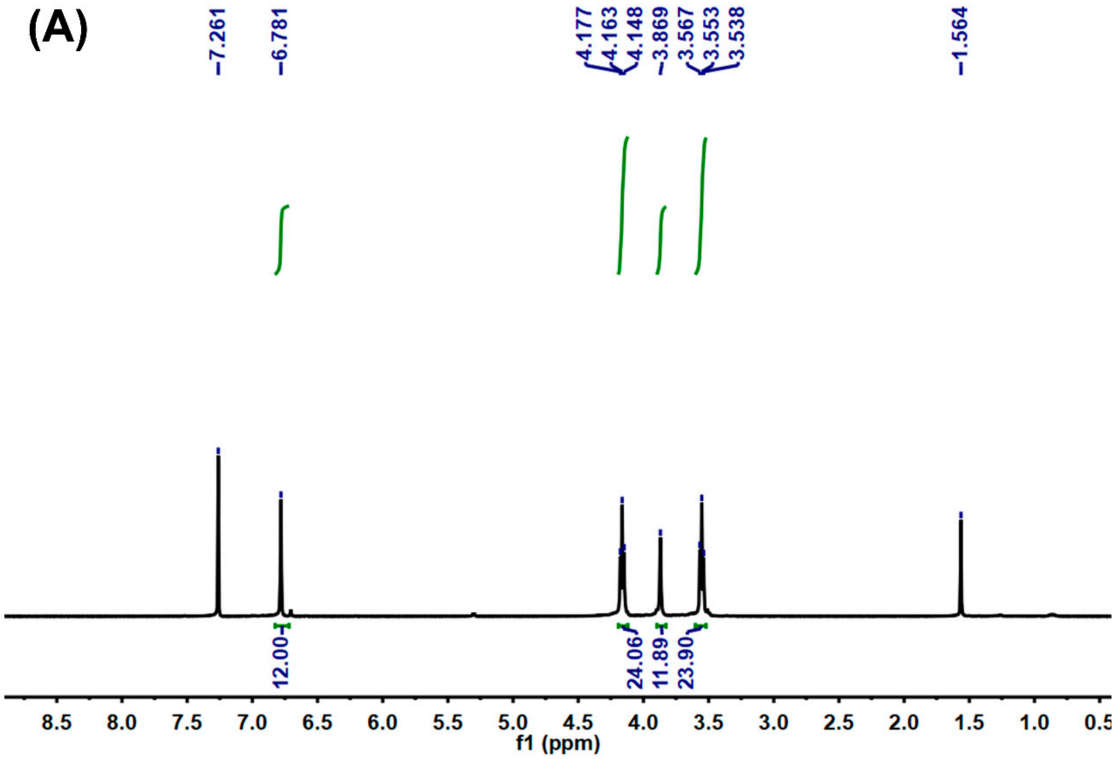

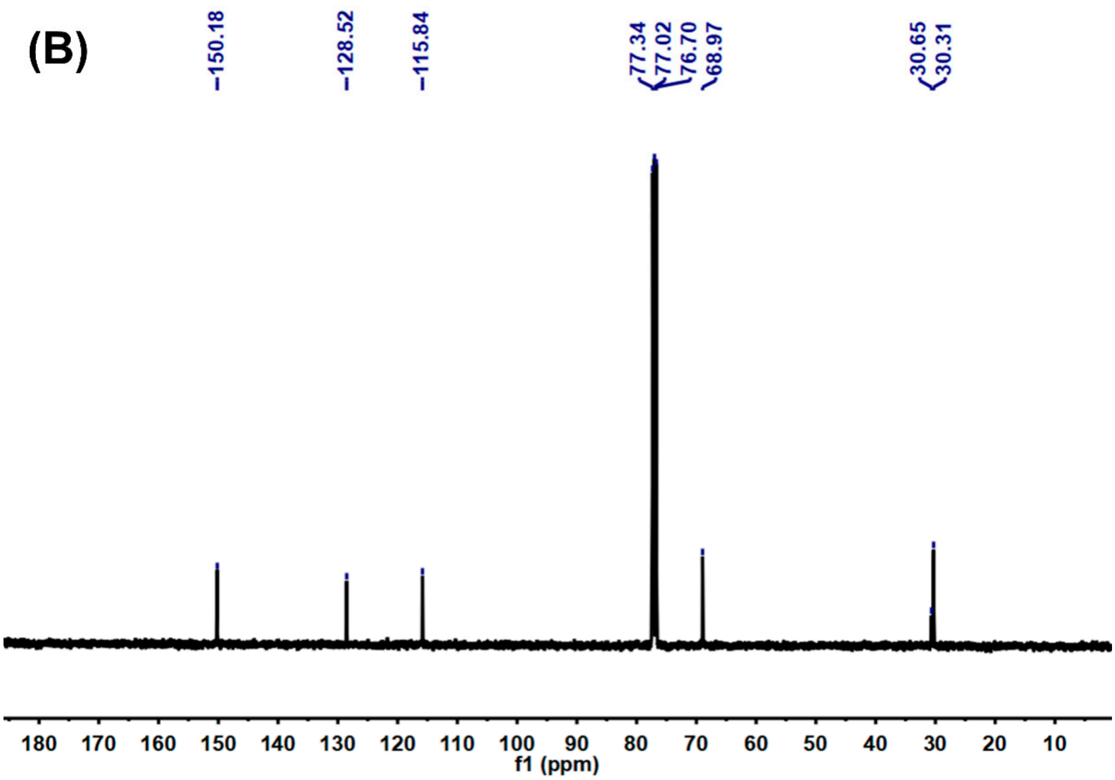

Figure S2. (**A**) ^{1}H NMR (600 MHz, chloroform-*d*, room temperature) and (**B**) ^{13}C NMR spectra (150 MHz, chloroform-*d*, room temperature) of Br6.

1.1.3. Synthesis of cPA6

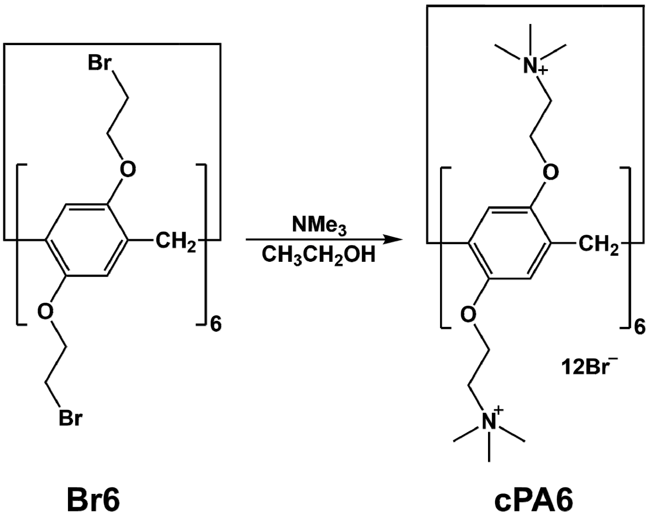

Scheme S4. Synthetic route to cPA6.

Br6 (1.00 g, 0.49 mmol) and trimethylamine (33 % in ethanol, 6.4 mL, 24 mmol) were added to ethanol of 50 mL. The solution was refluxed at 90 °C for 24 h. Then, the solvent was removed by evaporation, deionized water of 20 mL was added. After filtration, a clear solution was obtained. Then, the water was removed by evaporation to obtain cPA6 as a colorless solid (1.2 g, 90%). The ^{1}H NMR and ^{13}C NMR spectra of cPA6 are shown in Figure S3. ^{1}H NMR (600 MHz, D_2O, room temperature) δ (ppm): 6.82 (s, 12H), 4.42 (s, 24H), 3.87 (s, 12H), 3.67 (s, 24H), and 3.03 (s, 108H). ^{13}C NMR (150 MHz, D_2O, room temperature) δ (ppm): 149.85, 129.12, 116.28, 65.15, 63.43, 54.10, and 30.14.

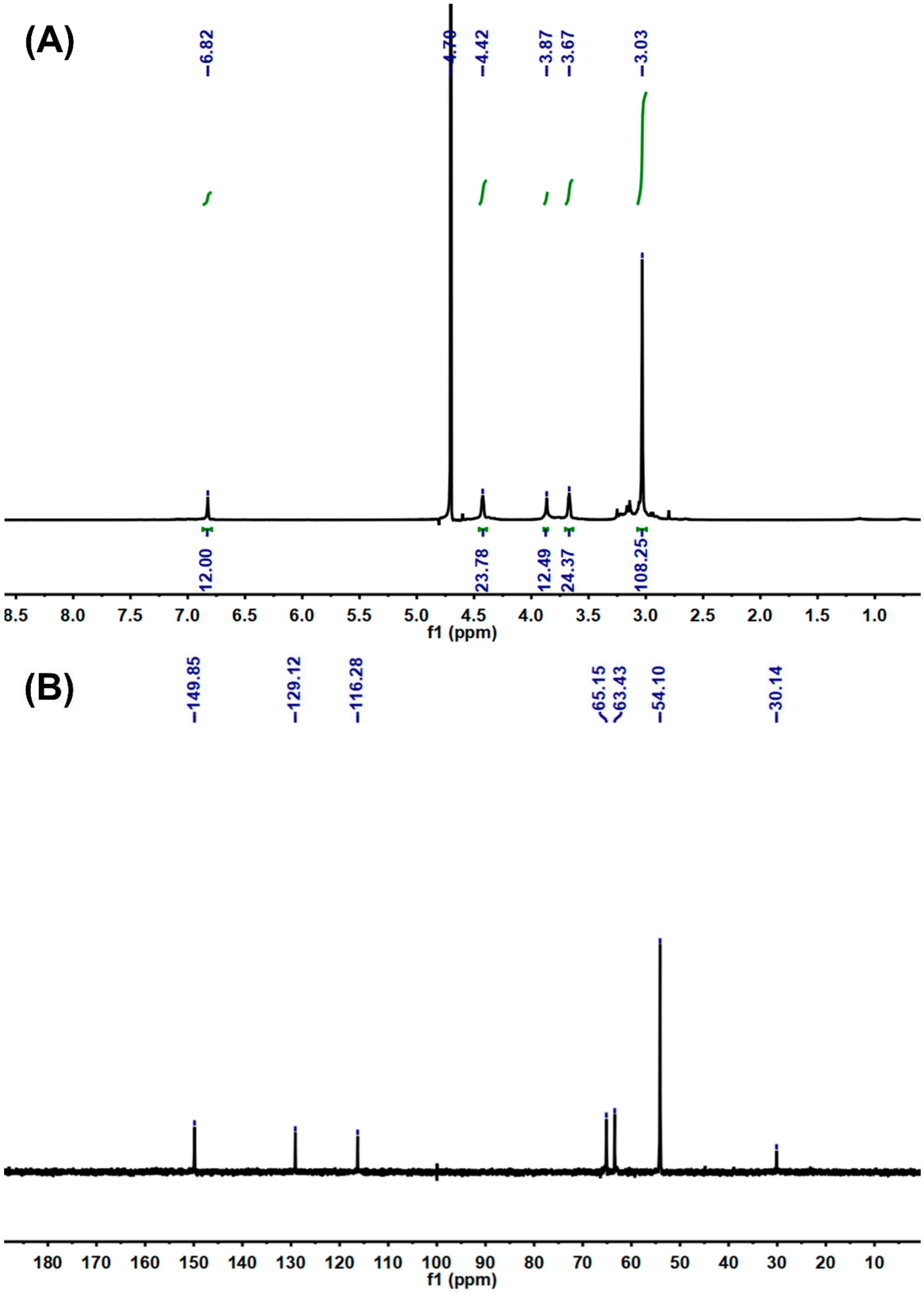

Figure S3. (**A**) ^1H NMR (600 MHz, D_2O, room temperature) and (**B**) ^{13}C NMR spectra (150 MHz, D_2O, room temperature) of cPA6.

1.2. Synthesis and characterization of cPA5

1.2.1. Synthesis of Br5

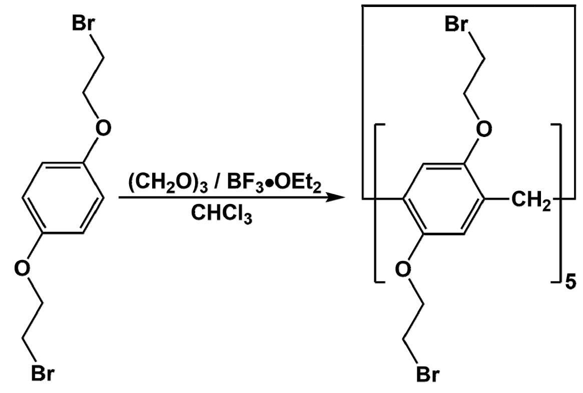

1 **Br5**

Scheme S5. Synthetic route to Br5.

To a solution of **1** (5.0 g, 15.4 mmol) in chloroform (300 mL), 1, 3, 5-trioxacyclohexane (0.9 g, 30.8 mmol) was added under nitrogen atmosphere. Then, boron trifluoride diethyl etherate ($BF_3{\cdot}OEt_2$, 4.38 g, 30.8 mmol) was added to the solution and the mixture was stirred at room temperature for 1 h. After the reaction was stopped by water and the solvent was removed, the obtained solid was purified by column chromatography on silica gel with petroleum ether/dichloromethane (1:2 v/v) as the eluent to obtain a white powder (2.2 g, 42 %). The ^{1}H NMR and ^{13}C NMR spectra of Br5 are shown in Figure S4. ^{1}H NMR (600 MHz, chloroform-*d*, room temperature) δ (ppm): 6.914 (s, 10H), 4.228 (t, J = 5.6 Hz, 20H), 3.843 (s, 10H), and 3.633 (t, J = 5.6 Hz, 20H). ^{13}C NMR (150 MHz, chloroform-*d*, room temperature) δ (ppm): 149.70, 129.10, 116.14, 69.01, 30.65, and 29.44.

(A)

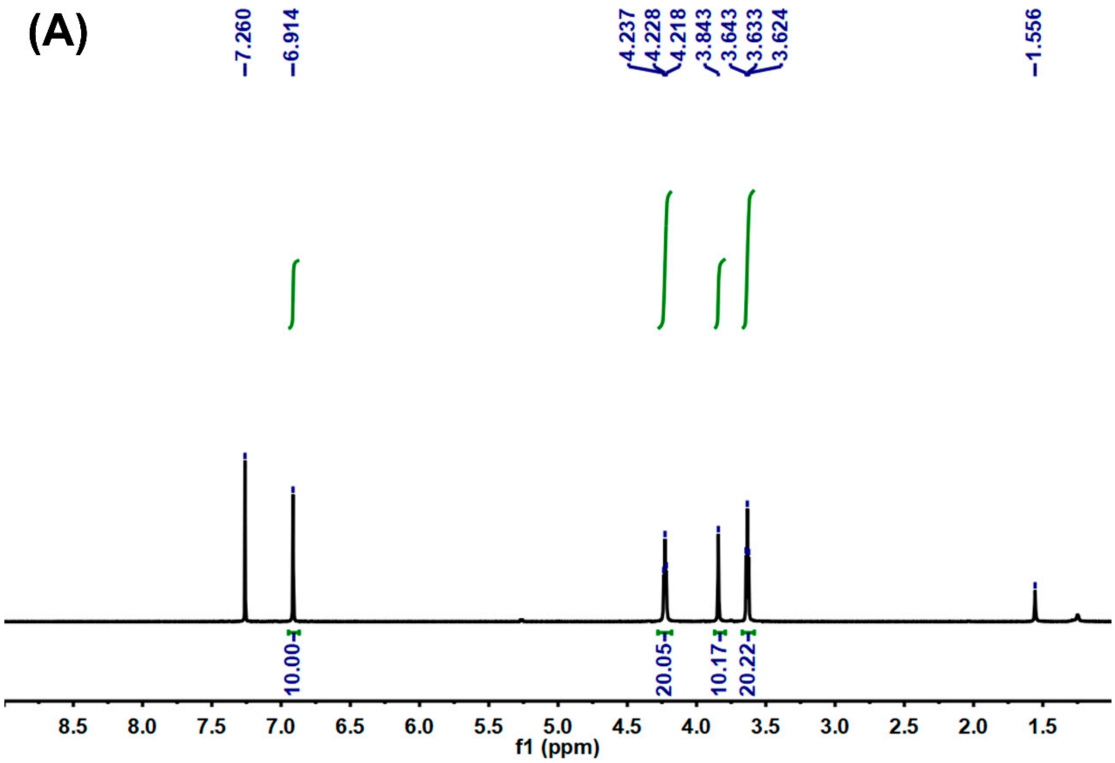

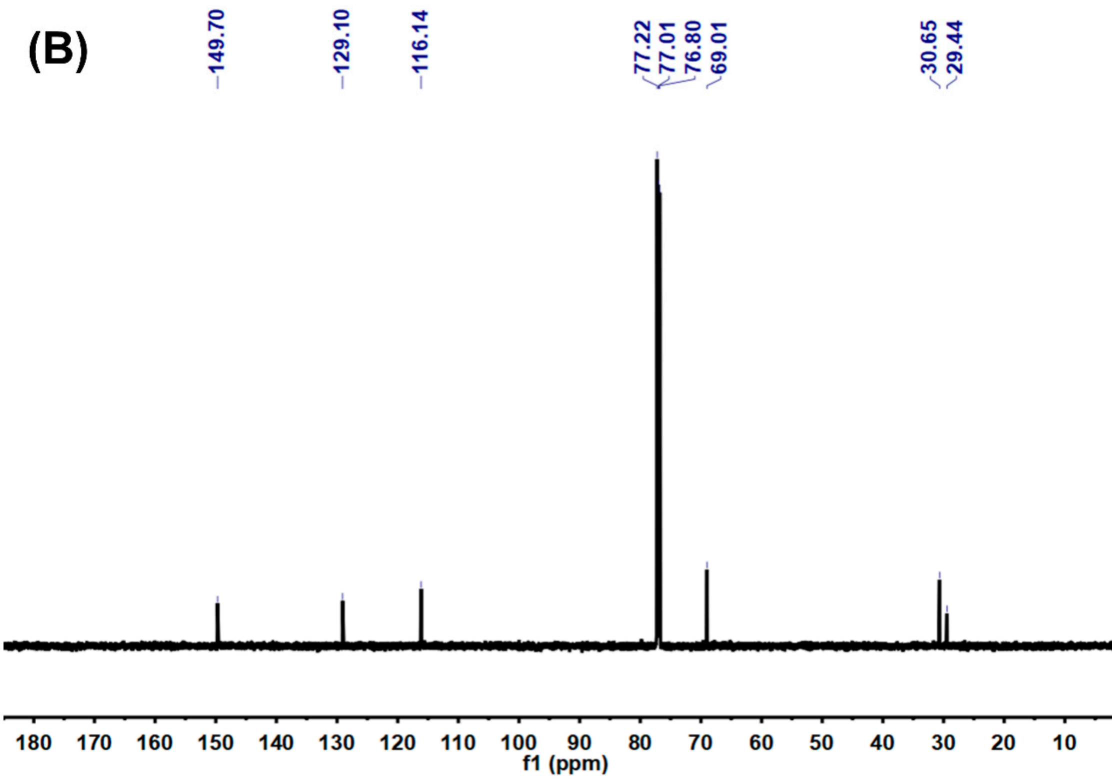

Figure S4. (**A**) ^{1}H NMR (600 MHz, chloroform-*d*, room temperature) and (**B**) ^{13}C NMR spectra (150 MHz, chloroform-*d*, room temperature) of Br5.

1.2.2. Synthesis of cPA5

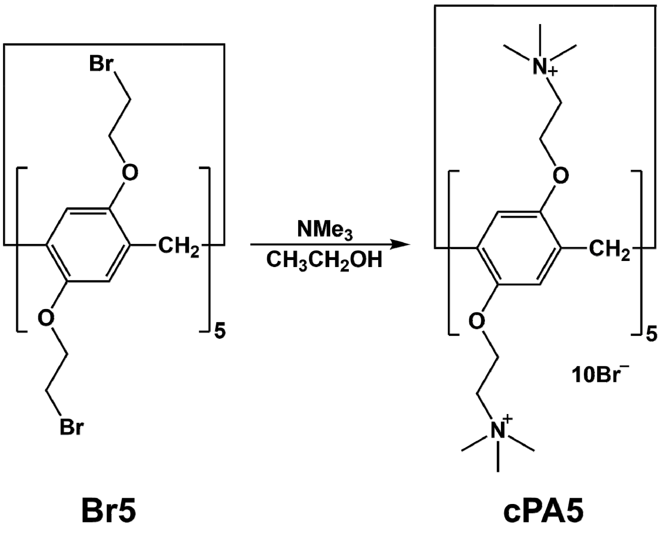

Scheme S6. Synthetic route to cPA5.

Br5 (1.0 g, 0.59 mmol) and trimethylamine (33 % in ethanol, 6.4 mL, 24 mmol) were added to ethanol (50 mL). The solution was refluxed at 90 °C for 24 h. Then, the solvent was removed by evaporation, deionized water (20 mL) was added. After filtration, a clear solution was obtained. Then, the water was removed by evaporation to obtain cPA5 as a colorless solid (1.2 g, 90 %). The ^{1}H NMR and ^{13}C NMR spectra of cPA5 are shown in Figure S5. ^{1}H NMR (600 MHz, D_2O, room temperature) δ (ppm): 6.83 (s, 10H), 4.34 (s, 20H), 3.82 (s, 10H), 3.70 (s, 20H), and 3.10 (s, 90H). ^{13}C

NMR (150 MHz, D_2O, room temperature) δ (ppm): 149.46, 130.02, 116.62, 64.98, 63.58, 54.16, and 29.64.

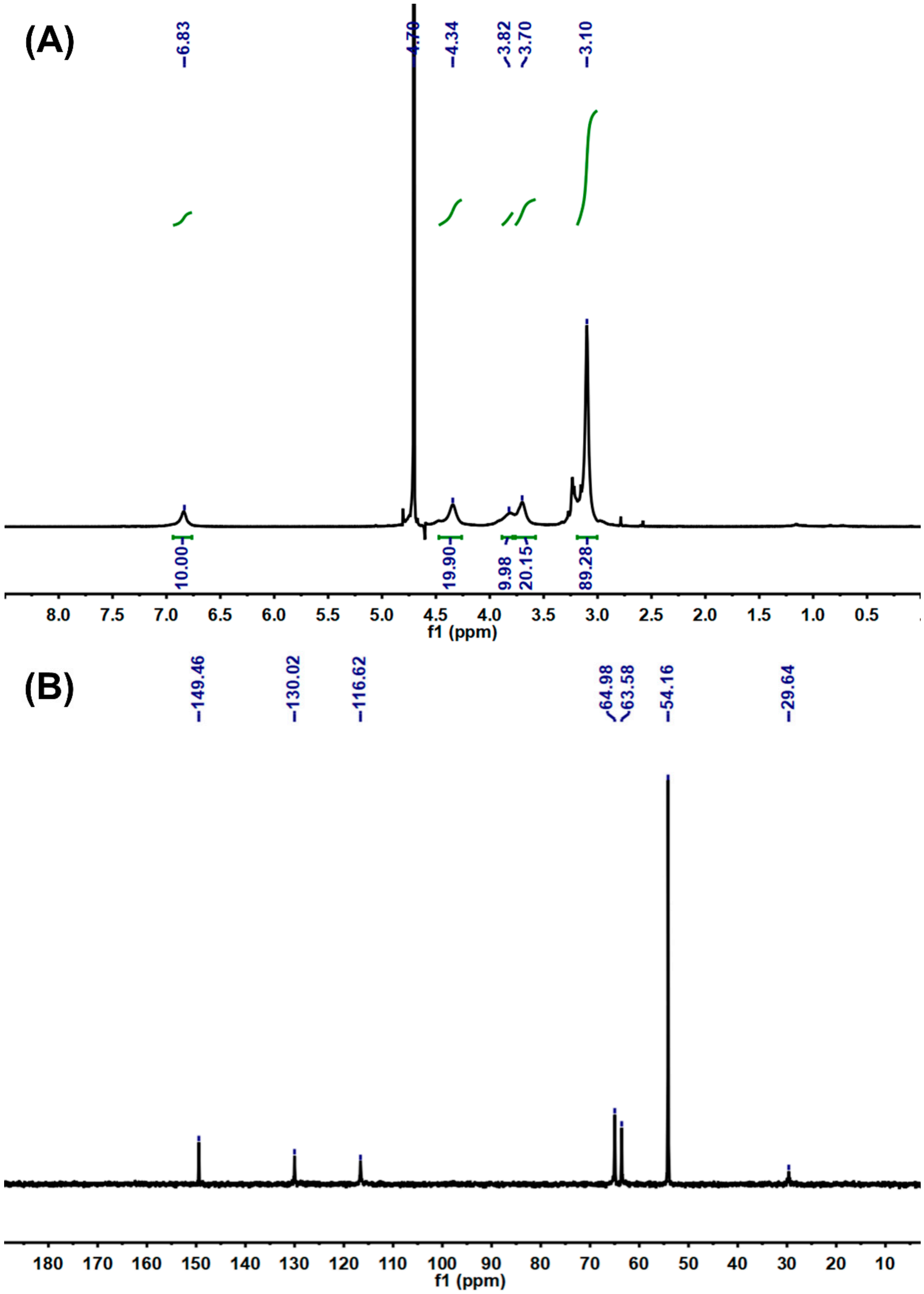

Figure S5. (**A**) ^{1}H NMR (600 MHz, D_2O, room temperature) and (**B**) ^{13}C NMR spectra (150 MHz, D_2O, room temperature) of cPA5.

1.3. Synthesis of wPA5

The water-soluble carboxylated pillar[5]arene (wPA5) was synthesized according to a published work [3-4] and its synthetic route is shown in Scheme S7.

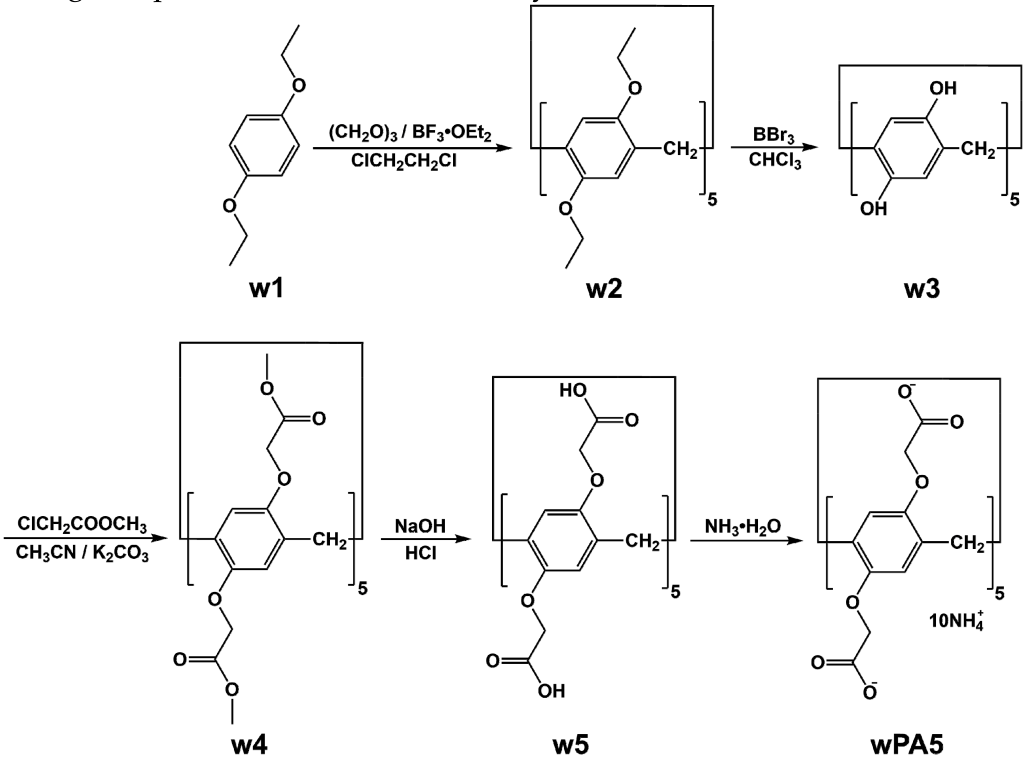

Scheme S7. Synthetic route to wPA5.

1.3.1. Synthesis of w2

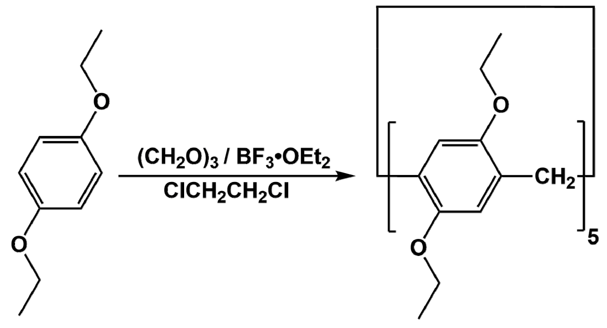

Scheme S8. Synthetic route to **w2**.

To a solution of w1 (1,4-diethoxybenzene, 5.7 g, 34.5 mmol) in 1,2-dichloroethane (100 mL), 1,3,5-trioxacyclohexane (1.1 g, 34.5 mmol) was added. Then, boron trifluoride diethyl etherate ($BF_3{\cdot}OEt_2$, 4.2 g, 30.8 mmol) was slowly added to the solution and the mixture was stirred at room temperature for 3 h. After the reaction was stopped by water, the organic phase was extracted by dichloromethane and dried by anhydrous sodium sulfate. The crude product was purified by column chromatography on silica gel with petroleum ether/acetic ether (30:1 v/v) as the eluent to obtain a white powder w2 (2.0 g, 50 %). The ^1H NMR and ^{13}C NMR spectra of w2 are shown in Figure S6. ^1H NMR (400 MHz, chloroform-*d*, room temperature) δ (ppm): 6.722 (s, 10H), 3.852 (q,

J=6.8Hz, 20H), 3.765 (s, 10H), and 1.261 (t, J=6.8Hz, 30H). ^{13}C NMR (100 MHz, chloroform-*d*, room temperature) δ (ppm): 149.84, 128.50, 115.14, 64.98, 63.80, 29.87, and 15.06.

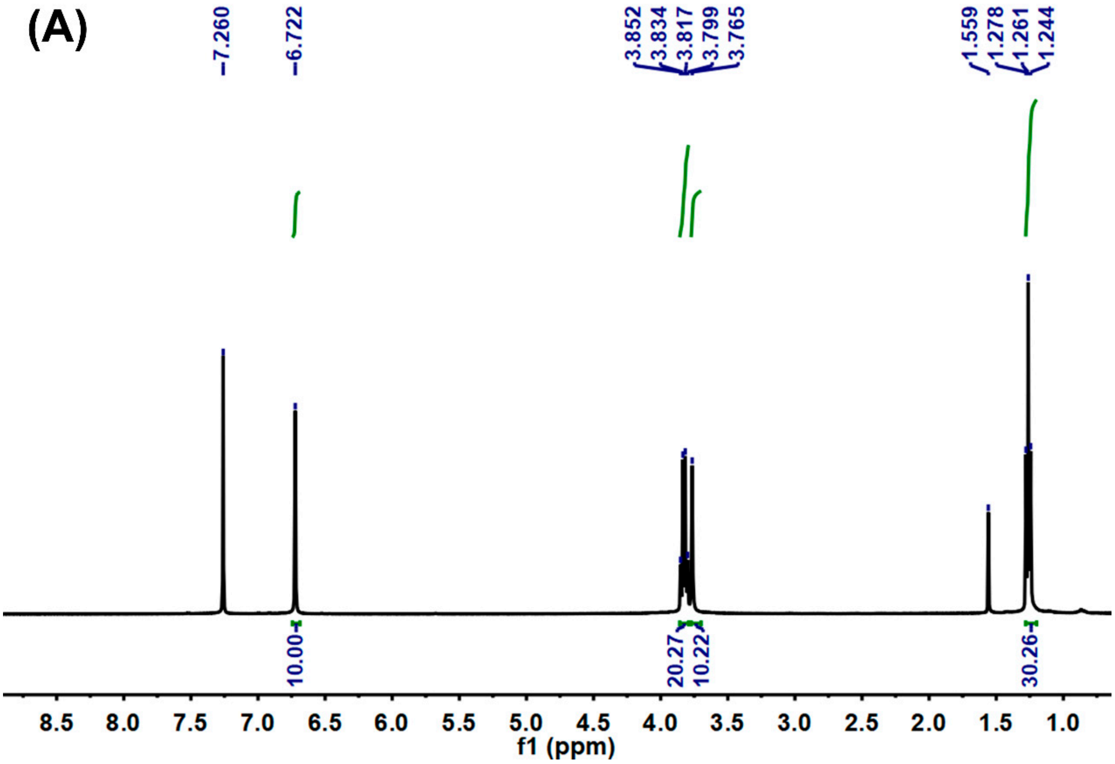

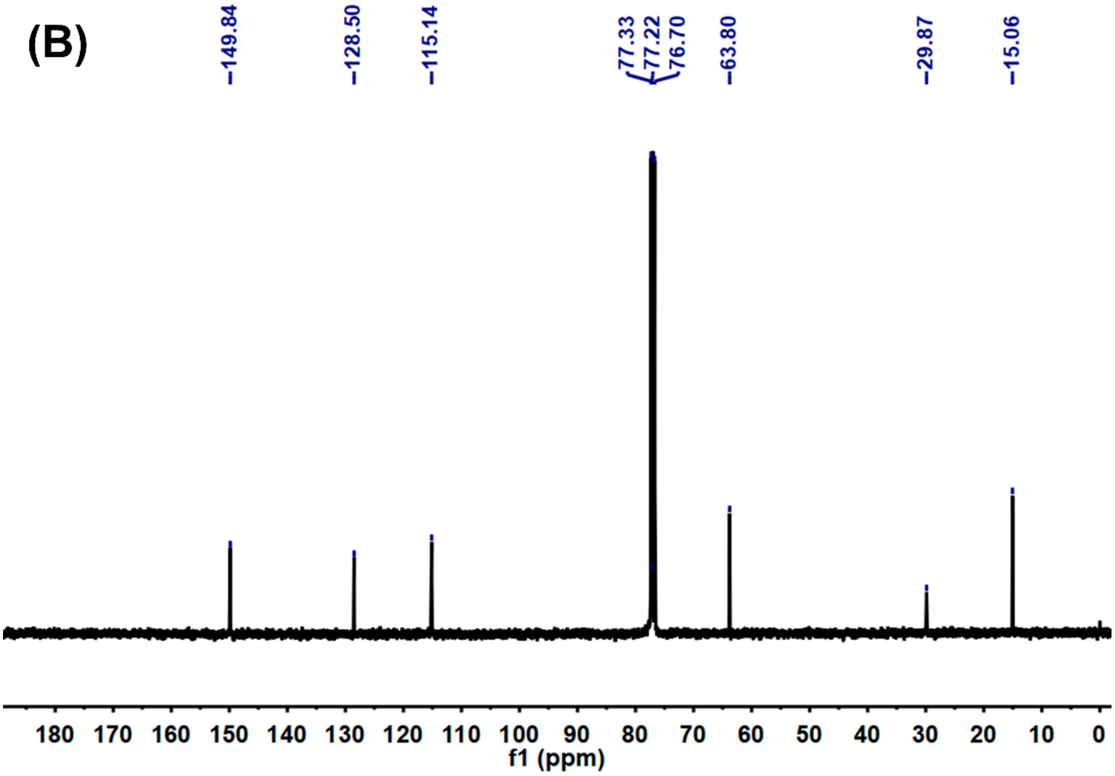

Figure S6. (**A**) ^{1}H NMR spectrum (400 MHz, chloroform-*d*, room temperature) and (**B**) ^{13}C NMR (100 MHz, chloroform-*d*, room temperature) of w2.

1.3.2. Synthesis of w3

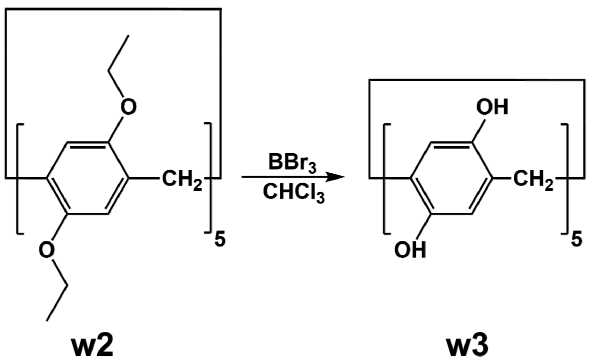

Scheme S9. Synthetic route to w3.

w2 (2.0 g, 2.2 mmol) was dissolved in chloroform (50 mL). Boron tribromide (4.5 mL, 44 mmol) was slowly added to the solution in ice bath and the mixture was stirred at room temperature for 24 h. After the reaction was stopped by water, the solvent was removed and a white powder w3 was obtained. The ^{1}H NMR and ^{13}C NMR spectra of w3 are shown in Figure S7. ^{1}H NMR (400 MHz, DMSO-d_6, room temperature) δ (ppm): 8.449 (s, 10H), 6.580 (s, 20H), and 3.432 (s, 10H). ^{13}C NMR (100 MHz, DMSO-d_6, room temperature) δ (ppm): 146.55, 126.91, 117.77, and 29.62.

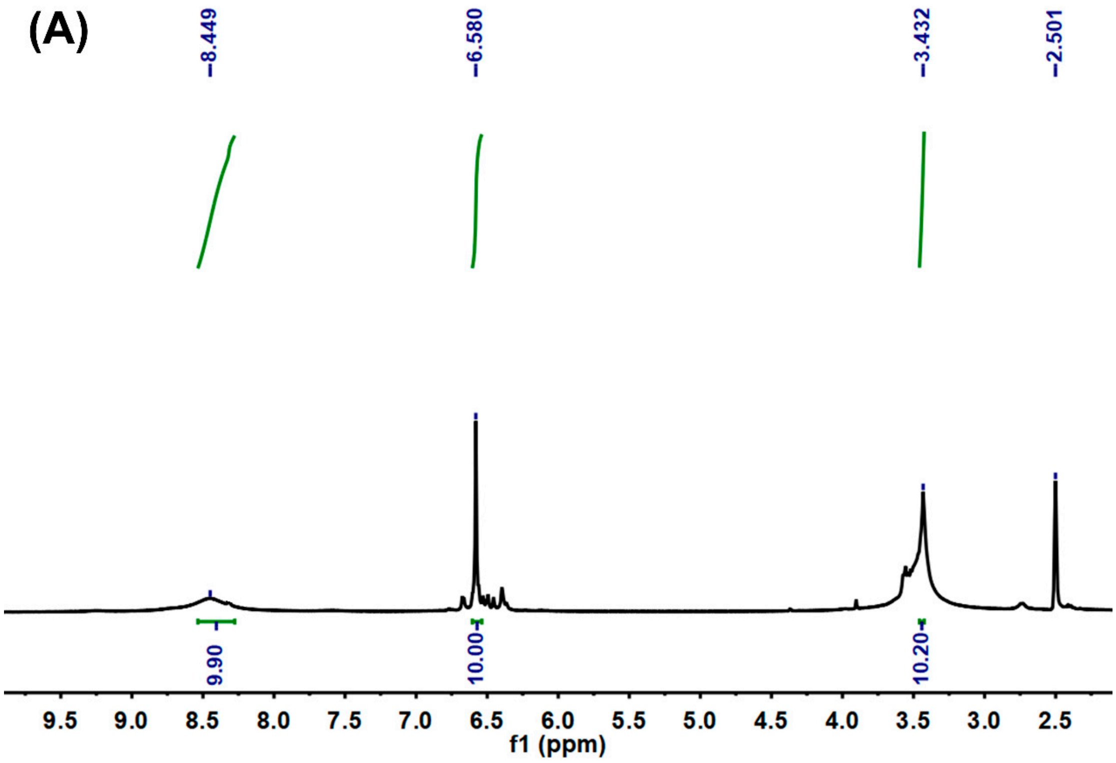

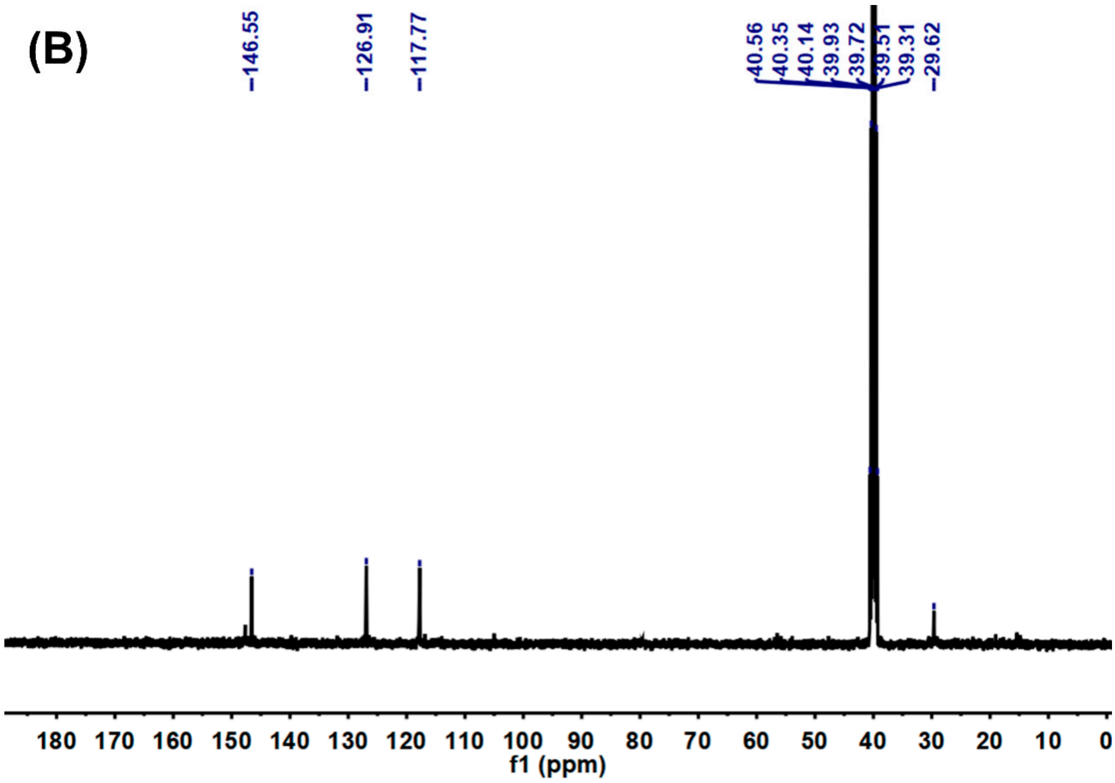

Figure S7. (A) ^{1}H NMR (400 MHz, DMSO-d_6, room temperature) and (B) ^{13}C NMR spectra (100 MHz, DMSO-d_6, room temperature) of w3.

1.3.3. Synthesis of w4

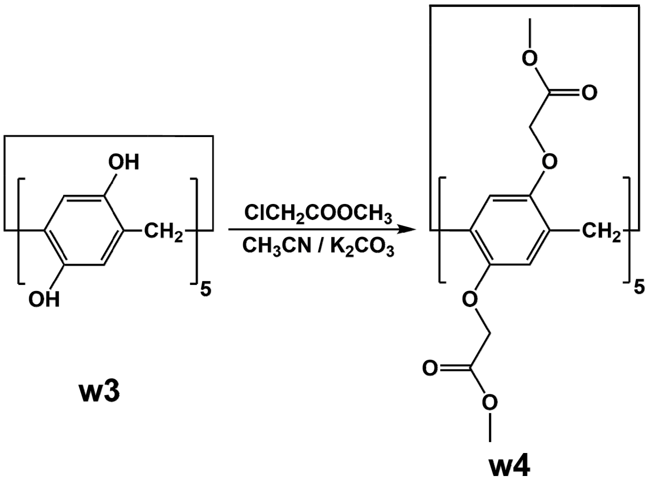

Scheme S10. Synthetic route to w4.

w3 (1.22 g, 2 mmol), anhydrous potassium carbonate (3.31 g, 24 mmol) and methyl chloroacetate were added to dry acetonitrile (50 mL). The solution was refluxed at 80 °C for 24 h under N_2. Then, the solvent was removed by evaporation, methyl alcohol (20 mL) was added. After recrystallization, white solid w4 was obtained. The ^{1}H NMR and ^{13}C NMR spectra of w4 are shown in Figure S8. ^{1}H NMR (400 MHz, chloroform-*d*, room temperature) δ (ppm): 6.990 (s, 10H), 4.557 (s, 20H), 3.855 (s, 10H), and 3.542 (s, 30H). ^{13}C NMR (100 MHz, chloroform-*d*, room temperature) δ (ppm): 169.82, 148.95, 128.50, 114.51, 65.51, 51.75, and 29.33.

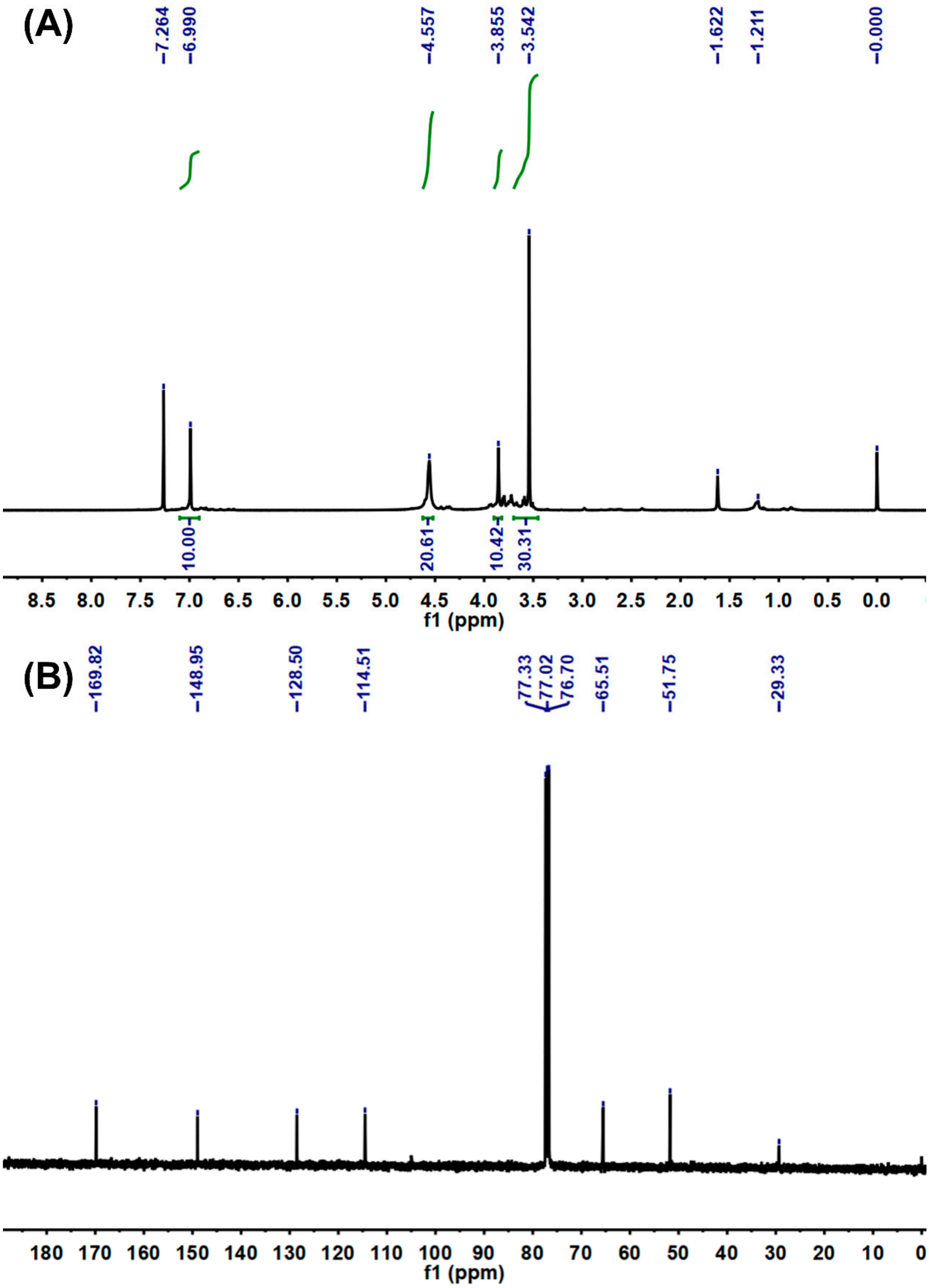

Figure S8. (A) ^{1}H NMR (400 MHz, chloroform-*d*, room temperature) and (B) ^{13}C NMR spectra (100 MHz, chloroform-*d*, room temperature) of w4.

1.3.4. Synthesis of w5

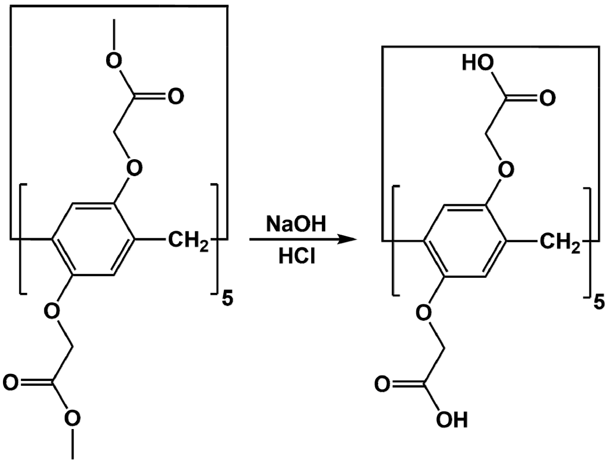

w4 w5

Scheme S11. Synthetic route to w5.

w4 (0.5 g, 0.37 mmol) and NaOH (0.3 g, 24 mmol) were added to deionized water (15 mL). The solution was refluxed at 90 °C for 24 h. Hydrochloric acid was added to the solution after cooling to room temperature and the white precipitate was obtained. After filtration, deionized water of 20 mL was added. Then, the water was removed by desiccation to obtain w5 of white solid. The ^{1}H NMR and ^{13}C NMR spectra of w5 are shown in Figure S9. ^{1}H NMR (400 MHz, chloroform-*d*, room temperature) δ (ppm): 12.948 (s, 10H), 7.109 (s, 10H), 4.675 (d, J=16Hz, 10H), 4.387 (d, J=15.6Hz, 10H), and 3.738 (s, 10H). ^{13}C NMR (100 MHz, chloroform-*d*, room temperature) δ (ppm): 170.90, 148.88, 128.50, 114.65, 65.48, and 29.05.

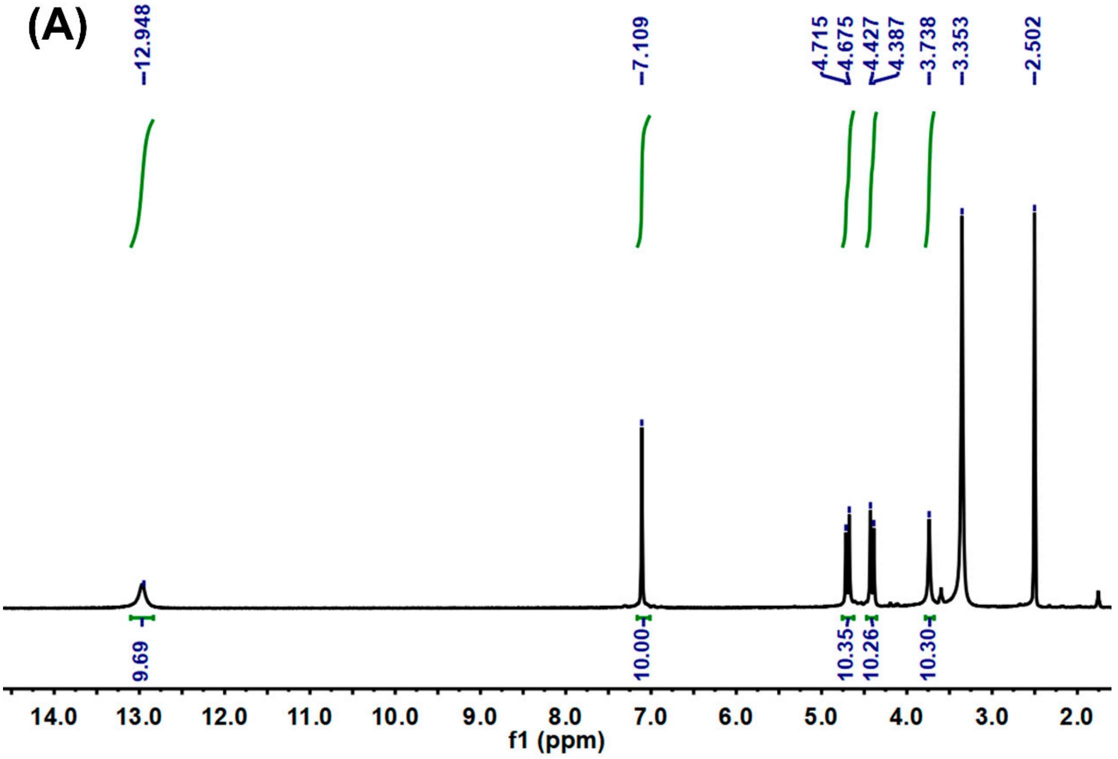

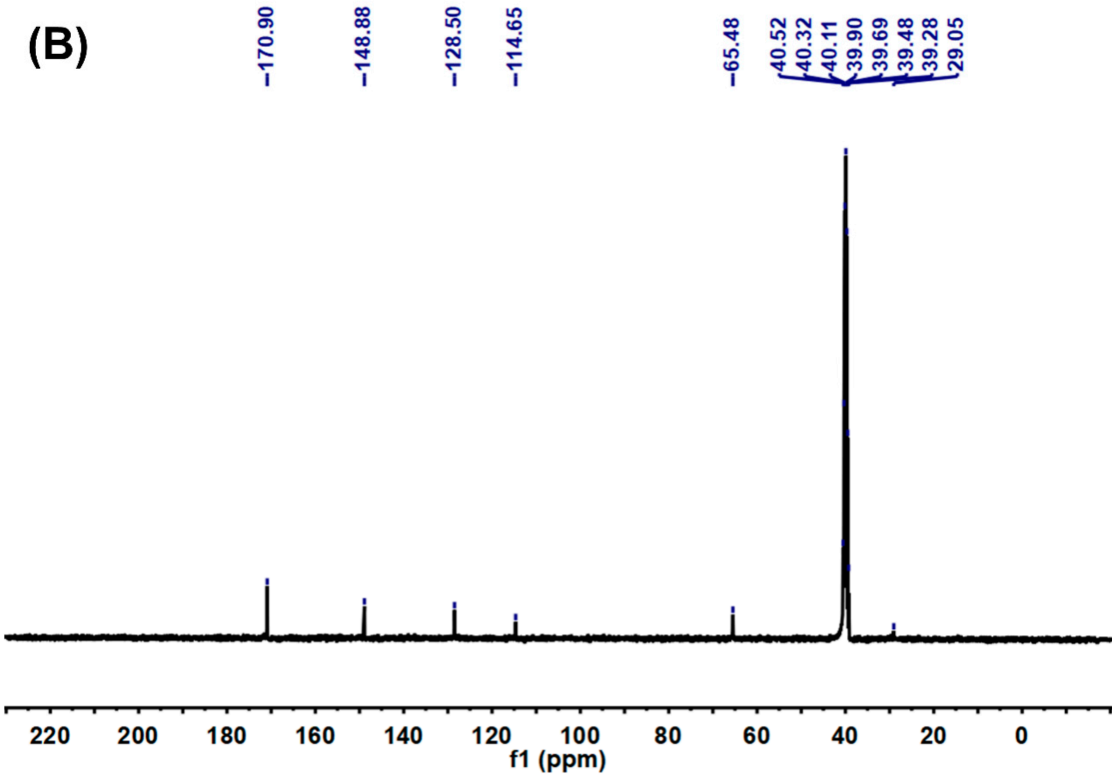

Figure S9. (A) ^{1}H NMR spectrum (400 MHz, DMSO-*d*6, room temperature) and (B) ^{13}C NMR (100 MHz, DMSO-*d*6, room temperature) of w5.

1.3.5. Synthesis of wPA5

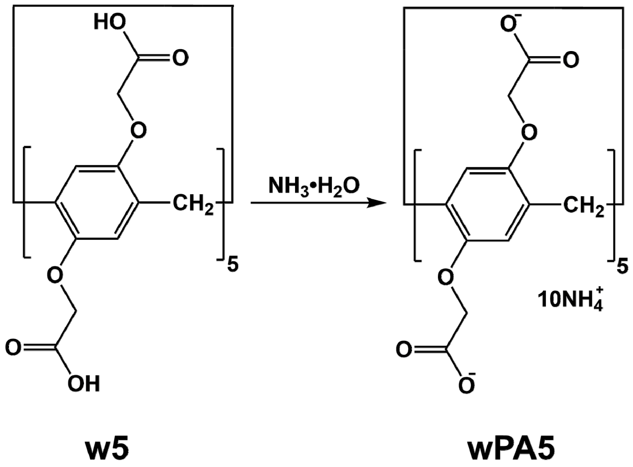

Scheme S12. Synthetic route to wPA5

Ammonia (61.4 g, 3.6 mmol) was added to the solution of w5 (400 mg, 0.336 mmol) in deionized water of 20 mL and the mixture was stirred at room temperature for 24 h. Then, the water was removed by evaporation to obtain wPA5 of colorless solid (400 mg, 95 %). The ^{1}H NMR and ^{13}C NMR spectra of wPA5 are shown in Figure S10. ^{1}H NMR (400 MHz, D_2O, room temperature) δ (ppm): 6.574 (s, 10H), 4.131 (s, 20H), and 3.701 (s, 10H). ^{13}C NMR (100 MHz, D_2O, room temperature) δ (ppm): 177.00, 149.36, 128.72, 114.94, 67.53, and 29.10.

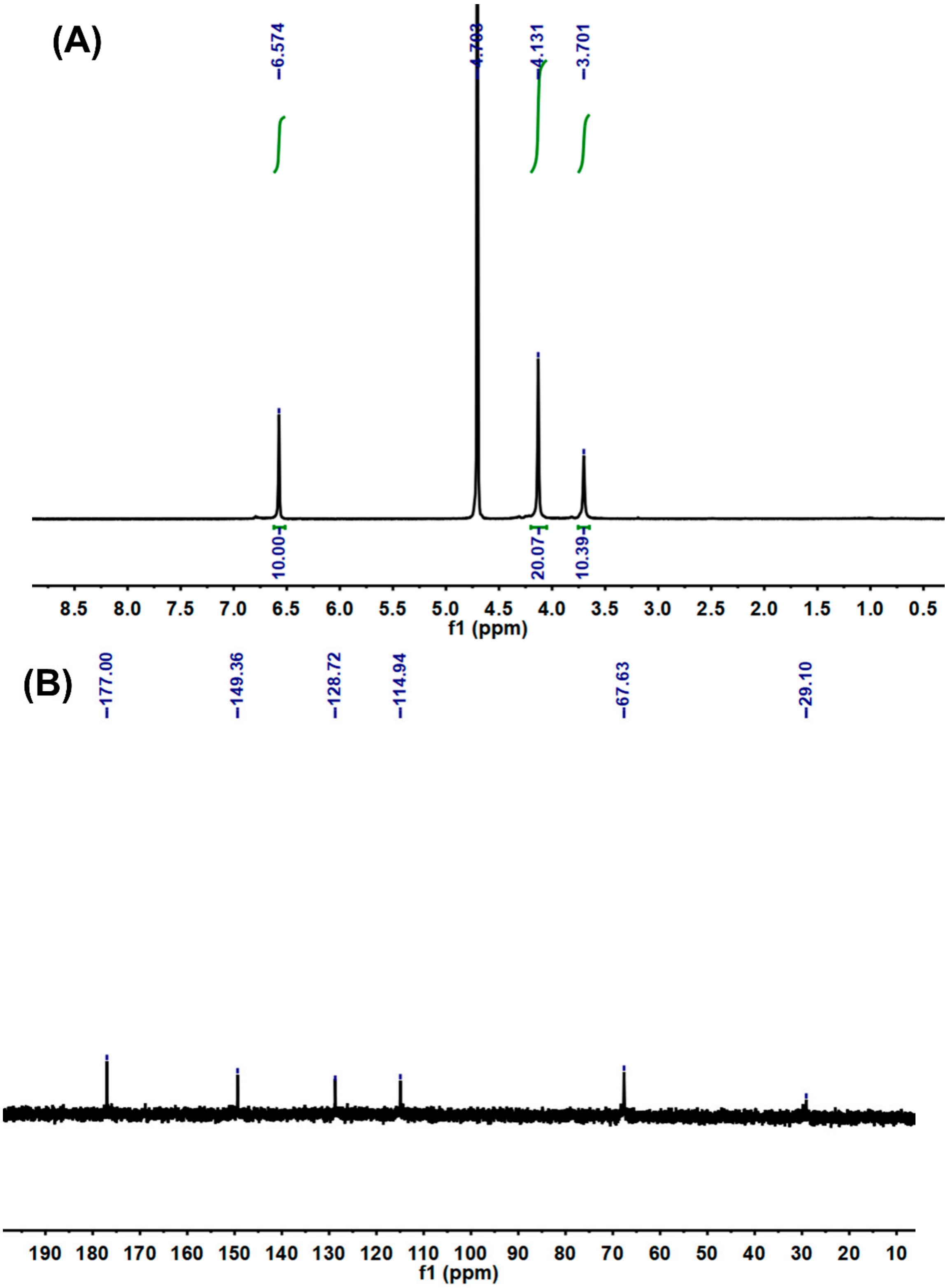

Figure S10. (**A**)^{1}H NMR (400 MHz, D_2O, room temperature) and (**B**) ^{13}C NMR spectra (100 MHz, D_2O, room temperature) of wPA5.

2. Characterization of host/guest complex

2.1. NMR spectroscopy of pEY

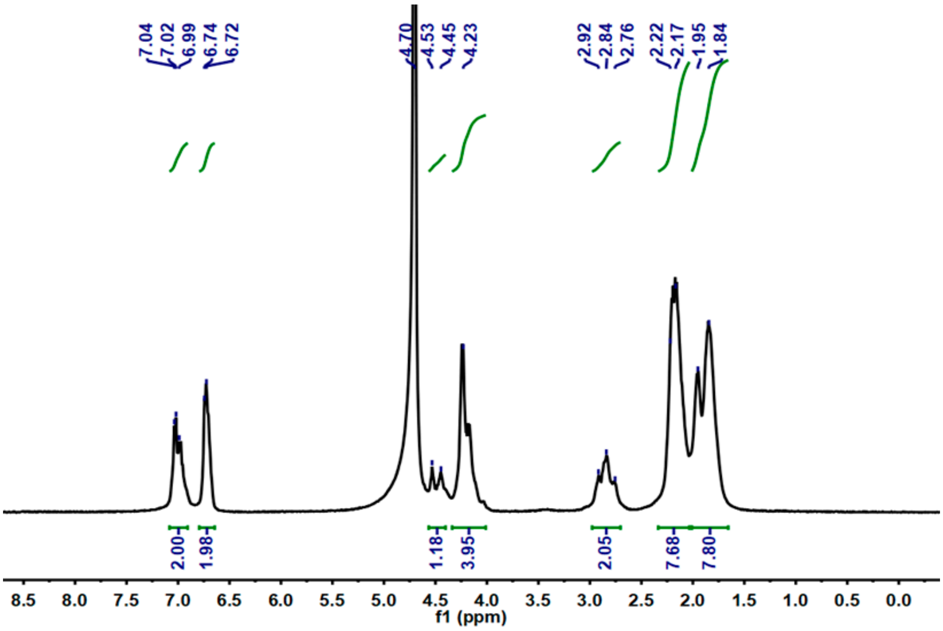

Figure S11. ^{1}H NMR spectrum (500 MHz, D_2O, room temperature) of pEY.

2.2. Isothermal titration calorimetry of pEY with cPA5

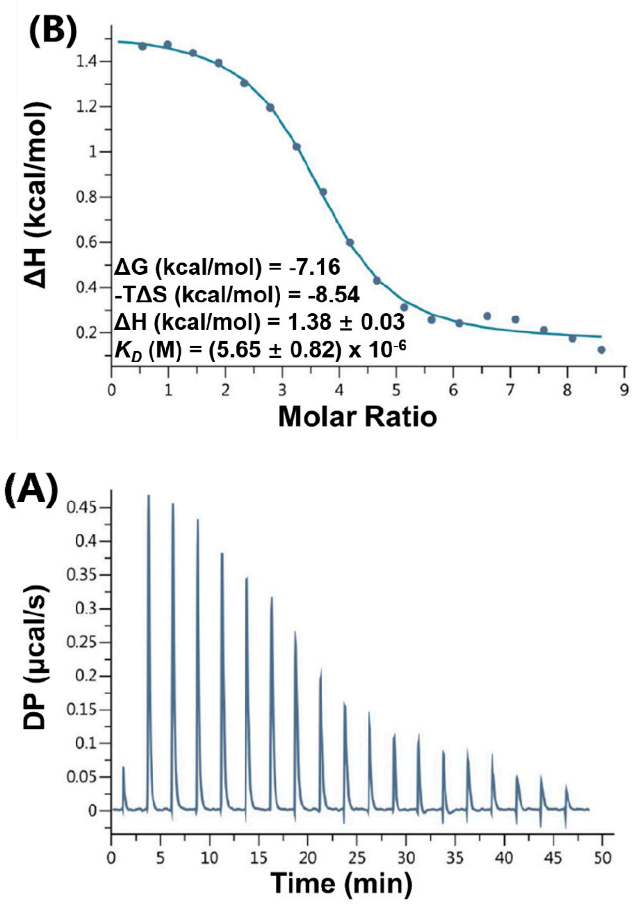

Figure S12. Microcalorimetric titration of cPA5 with pEY in PBS (pH 7.2) at 298.15 K. (A) Raw ITC data for 19 sequential injections (10 µL per injection) of a pEY solution (2.00 mM) into a cPA5 solution (0.045 mM). (B) Net reaction heat obtained from the integration of the calorimetric traces.

2.3. Fluorescence spectrum of cPA6 with pEY

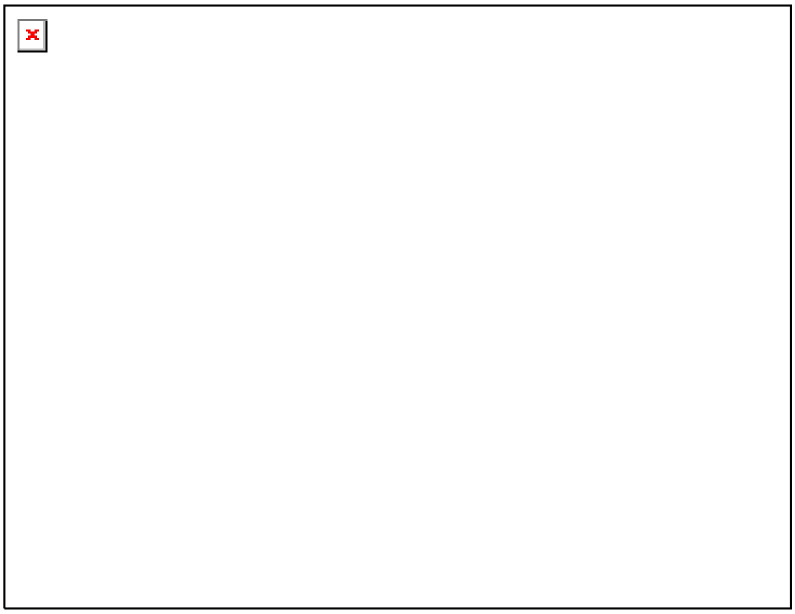

Figure S13. Fluorescence spectra of cPA6 (0.1 mM) in PBS (pH 7.2) at 25 ^{o}C with different concentrations of pEY ranging from 0 to 55 μM. λexc = 276 nm.

2.4. NMR spectroscopy of host/guest complex

For the preparation of Tris-DCl buffered D_2O: Tris(hydroxymethyl-d_3)amino-d_2- methane (29.7 mg) was dissolved in D_2O of 3.0 mL to obtain tris-d_{11} solution (75 mM); pD was adjusted to 7.20 by addition of small amounts of DCl, until the pH reading was 6.80. The account of pD was calculated by the following equation:

pD = pH* + 0.40 (where pH* is the pH reading of D_2O solution).[5-6]

3. Effect of BPNS@ cPA6 on uptake and cell apoptosis

3.1. Uptake of BPNS@cPA6 in cells

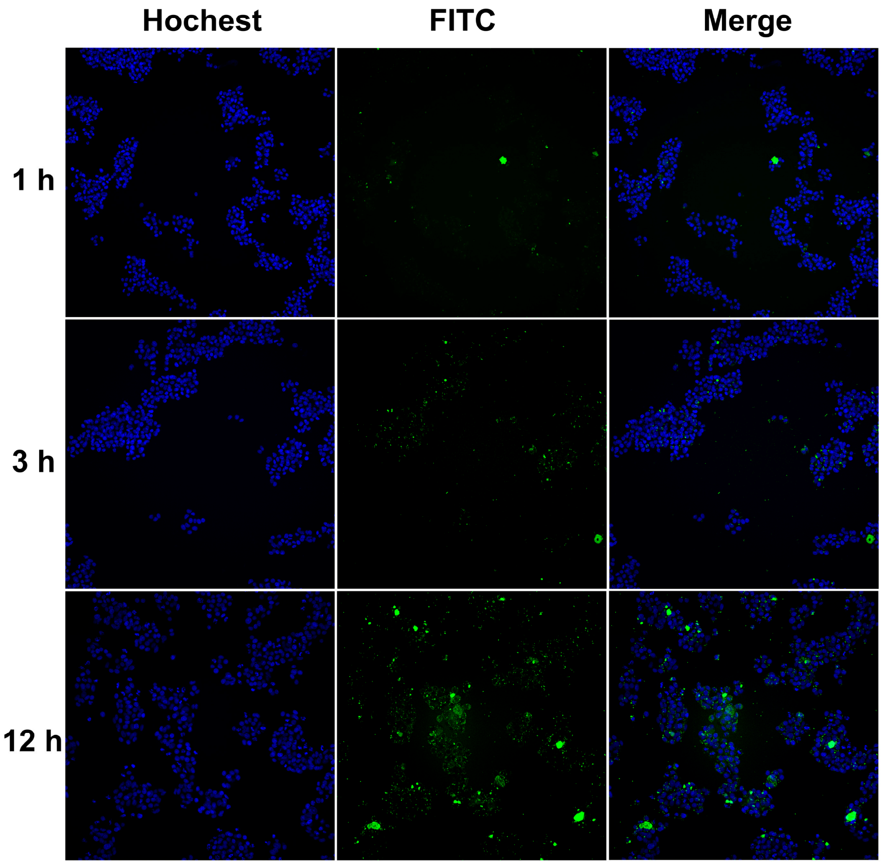

Figure S14. Fluorescence images of HepG2 cells incubated with BPNS@cPA6 for different times.

3.2. Cell apoptosis

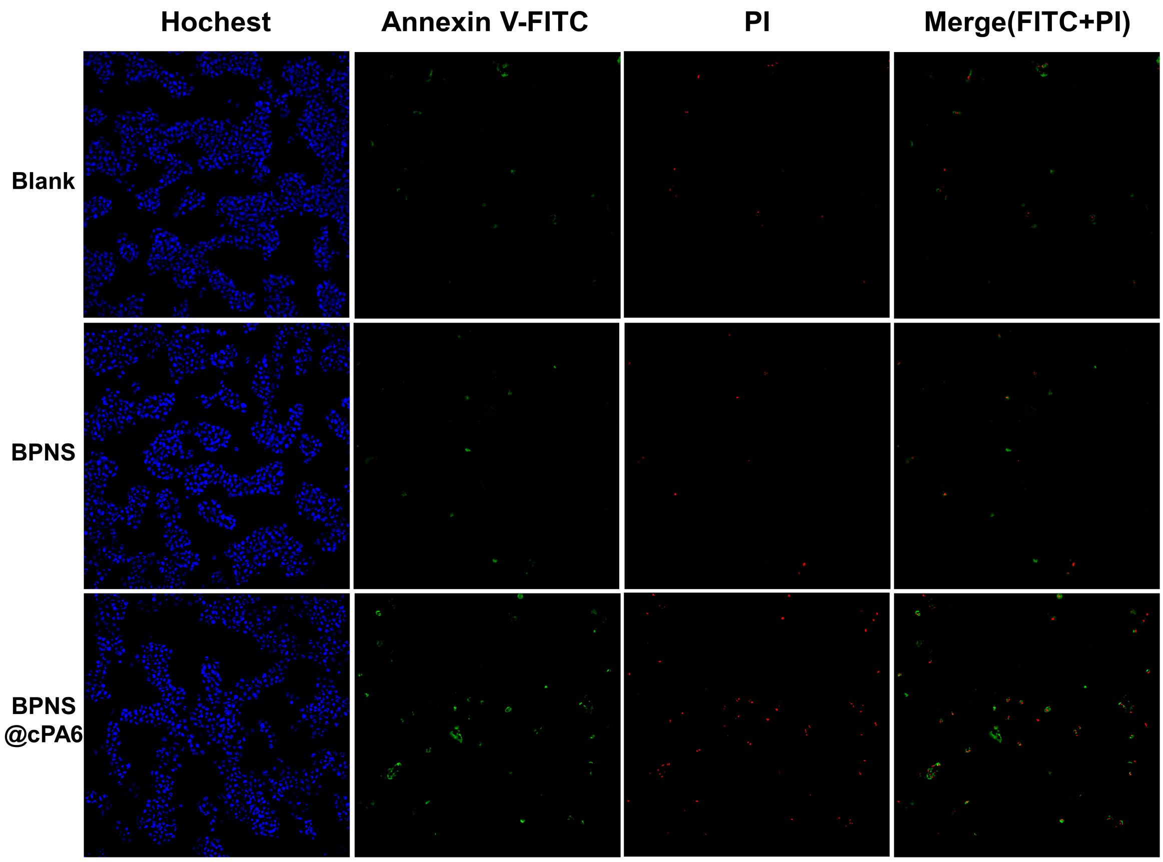

Figure S15. Fluorescence images of HepG2 cells incubated with PBS, BPNS and BPNS@cPA6 by Cell Apoptosis Detection Kit for 24 h.

References

1. Ma, Y.; Ji, X.; Xiang, F.; Chi, X.; Han, C.; He, J.; Abliz, Z.; Chen, W.; Huang, F., A cationic water-soluble pillar[5]arene: synthesis and host–guest complexation with sodium 1-octanesulfonate. *Chemical Communications* **2011,** 47, (45), 12340-12342, doi:10.1039/C1CC15660H.
2. Ogoshi, T.; Hashizume, M.; Yamagishi, T.; Nakamoto, Y., Synthesis, conformational and host–guest properties of water-soluble pillar[5]arene. *Chemical Communications* **2010,** 46, (21), 3708-3710, doi:10.1039/C0CC00348D.
3. Covington, A. K.; Paabo, M.; Robinson, R. A.; Bates, R. G., Use of the glass electrode in deuterium oxide and the relation between the standardized pD (paD) scale and the operational pH in heavy water. *Analytical Chemistry* **1968,** 40, (4), 700-706, doi:10.1021/ac60260a013.
4. Joseph, R.; Naugolny, A.; Feldman, M.; Herzog, I. M.; Fridman, M.; Cohen, Y., Cationic pillararenes potently inhibit biofilm formation without affecting bacterial growth and viability. *Journal of the American Chemical Society* **2016,** 138, (3), 754-757, doi:10.1021/jacs.5b11834.
5. Li, C.; Shu, X.; Li, J.; Chen, S.; Han, K.; Xu, M.; Hu, B.; Yu, Y.; Jia, X., Complexation of 1,4-bis(pyridinium)butanes by negatively charged carboxylatopillar[5]arene. *Journal of Organic Chemistry* **2011,** 76, (20), 8458-8465, doi:10.1021/jo201185e.
6. Baumann, E. W., Determination of deuterium ion concentration in dilute unbuffered deuterium oxide solutions. *Analytical Chemistry* **1966,** 38, (9), 1255-1257, doi:10.1021/ac60241a036.
